# Asynchrony among local communities stabilises ecosystem function of metacommunities

**DOI:** 10.1111/ele.12861

**Published:** 2017-10-24

**Authors:** Kevin R. Wilcox, Andrew T. Tredennick, Sally E. Koerner, Emily Grman, Lauren M. Hallett, Meghan L. Avolio, Kimberly J. La Pierre, Gregory R. Houseman, Forest Isbell, David Samuel Johnson, Juha M. Alatalo, Andrew H. Baldwin, Edward W. Bork, Elizabeth H. Boughton, William D. Bowman, Andrea J. Britton, James F. Cahill, Scott L. Collins, Guozhen Du, Anu Eskelinen, Laura Gough, Anke Jentsch, Christel Kern, Kari Klanderud, Alan K. Knapp, Juergen Kreyling, Yiqi Luo, Jennie R. McLaren, Patrick Megonigal, Vladimir Onipchenko, Janet Prevéy, Jodi N. Price, Clare H. Robinson, Osvaldo E. Sala, Melinda D. Smith, Nadejda A. Soudzilovskaia, Lara Souza, David Tilman, Shannon R. White, Zhuwen Xu, Laura Yahdjian, Qiang Yu, Pengfei Zhang, Yunhai Zhang

**Affiliations:** ^1^ Department of Microbiology and Plant Biology University of Oklahoma 770 Van Vleet Oval Norman OK 73019 USA; ^2^ Department of Wildland Resources and the Ecology Center Utah State University 5230 Old Main Hill Logan UT 84321 USA; ^3^ Department of Biology University of North Carolina Greensboro Greensboro NC 27412 USA; ^4^ Biology Department Eastern Michigan University 441 Mark Jefferson Science Complex Ypsilanti MI 48197 USA; ^5^ Environmental Studies Program and Department of Biology University of Oregon Eugene OR 97403 USA; ^6^ Morton K. Blaustein Department of Earth and Planetary Sciences Johns Hopkins University 301 Olin Hall 3400 N. Charles Street Baltimore MD 21218 USA; ^7^ Smithsonian Environmental Research Center 647 Contees Wharf Road Edgewater MD 21037 USA; ^8^ Department of Biological Sciences Wichita State University Wichita KS 67260 USA; ^9^ Department of Ecology, Evolution and Behavior University of Minnesota Saint Paul MN 55108 USA; ^10^ Virginia Institute of Marine Science Gloucester Point VA 23062 USA; ^11^ Department of Biological and Environmental Sciences Qatar University Doha Qatar; ^12^ Department of Environmental Science and Technology University of Maryland College Park MD 20742 USA; ^13^ Agriculture/Forestry Center University of Alberta Edmonton Alberta Canada T6G 2P5; ^14^ Archbold Biological Station MacArthur Agroecology Research Center 300 Buck Island Ranch Road Lake Placid FL 33852 USA; ^15^ Department of Ecology and Evolutionary Biology and Mountain Research Station University of Colorado Boulder CO 80309 USA; ^16^ The James Hutton Institute Craigiebuckler Aberdeen AB15 8QH UK; ^17^ Department of Biological Sciences University of Alberta Edmonton AB T6G 2E9 Canada; ^18^ Department of Biology University of New Mexico Albuquerque NM 87131 USA; ^19^ School of Life Science Lanzhou University Lanzhou Gansu China; ^20^ Department of Physiological Diversity Helmholtz Center for Environmental Research – UFZ Permoserstr. 15 D‐04318 Leipzig Germany; ^21^ German Centre for Integrative Biodiversity Research (iDiv) Halle‐Jena‐ Leipzig Deutscher Platz 5e D‐04103 Leipzig Germany; ^22^ Department of Ecology University of Oulu P.O. Box 3000 FI‐90014 Oulu Finland; ^23^ Department of Biological Sciences Towson University Towson MD 21252 USA; ^24^ Department of Disturbance Ecology University of Bayreuth D‐95440 Bayreuth Germany; ^25^ Northern Research Station US Forest Service 5985 Highway K Rhinelander WI 54501 USA; ^26^ Faculty of Environmental Sciences and Natural Resource Management Norwegian University of Life Sciences P.O. Box 5003 NO‐1432 Aas Norway; ^27^ Department of Biology Graduate Degree Program in Ecology Colorado State University Fort Collins CO 80523 USA; ^28^ Institute of Botany and Landscape Ecology Experimental Plant Ecology Greifswald University Soldmannstrasse 15 D‐17487 Greifswald Germany; ^29^ Department of Biological Sciences Center for Ecosystem Science and Society (Ecoss) Northern Arizona University Flagstaff AZ 86011 USA; ^30^ Department for Earth System Science Tsinghua University Beijing China; ^31^ Department of Biological Sciences University of Texas at El Paso El Paso TX 79968 USA; ^32^ Smithsonian Environmental Research Center Edgewater MD 20754 USA; ^33^ Department of Geobotany Moscow State Lomonosov University Leninskie gory 1‐12 119234 Moscow Russia; ^34^ USFS Pacific Northwest Research Station 3625 93^rd^ Ave SW Olympia WA 98512 USA; ^35^ Institute of Land, Water and Society Charles Sturt University Albury NSW 2640 Australia; ^36^ School of Earth & Environmental Sciences The University of Manchester Williamson Building, Oxford Road Manchester M13 9PL UK; ^37^ School of Life Sciences and School of Sustainability Arizona State University Tempe AZ 85287 USA; ^38^ Conservation Biology Department Institute of Environmental Sciences CML, Leiden University Einsteinweg 2 2333 CC Leiden The Netherlands; ^39^ Oklahoma Biological Survey University of Oklahoma Norman OK 73019 USA; ^40^ Department of Ecology, Evolution and Behavior College of Biological Sciences University of Minnesota Saint Paul MN 55108 USA; ^41^ Environment and Parks Government of Alberta Edmonton AB T5K 2M4 Canada; ^42^ Institute of Applied Ecology Chinese Academy of Sciences Shenyang Liaoning 110016 China; ^43^ Universidad de Buenos Aires Consejo Nacional de Investigaciones Científicas y Técnicas Instituto de Investigaciones Fisiológicas y Ecológicas Vinculadas a la Agricultura (IFEVA) Facultad de Agronomía Buenos Aires Argentina; ^44^ National Hulunber Grassland Ecosystem Observation and Research Station/Institute of Agricultural Resources and Regional Planning Chinese Academy of Agricultural Sciences Beijing 100081 China; ^45^ State Key Laboratory of Vegetation and Environmental Change Institute of Botany Chinese Academy of Sciences Beijing 100093 China; ^46^ Department of Agroecology Aarhus University Blichers Allé 20 8830 Tjele Denmark

**Keywords:** Alpha diversity, alpha variability, beta diversity, biodiversity, CoRRE data base, patchiness, plant communities, primary productivity, species synchrony

## Abstract

Temporal stability of ecosystem functioning increases the predictability and reliability of ecosystem services, and understanding the drivers of stability across spatial scales is important for land management and policy decisions. We used species‐level abundance data from 62 plant communities across five continents to assess mechanisms of temporal stability across spatial scales. We assessed how asynchrony (i.e. different units responding dissimilarly through time) of species and local communities stabilised metacommunity ecosystem function. Asynchrony of species increased stability of local communities, and asynchrony among local communities enhanced metacommunity stability by a wide range of magnitudes (1–315%); this range was positively correlated with the size of the metacommunity. Additionally, asynchronous responses among local communities were linked with species’ populations fluctuating asynchronously across space, perhaps stemming from physical and/or competitive differences among local communities. Accordingly, we suggest spatial heterogeneity should be a major focus for maintaining the stability of ecosystem services at larger spatial scales.

## Introduction

Ecosystem stability through time provides information about an ecosystem's ability to maintain consistent interannual functioning despite variations in environmental conditions and disturbance (Turner *et al*. 1993; Tilman *et al*. 2006). Conservation managers seek stability because it suggests sustainability of species, community and ecosystem functioning (White & Jentsch 2001). Understanding the mechanisms that control ecosystem stability can help guide management actions aimed at making ecosystem functioning more stable, and thus predictable, through time (Fisher *et al*. 2009). Many previous approaches to identifying mechanisms underlying stability have focused on relatively small spatial scales (i.e. study plots) (Tilman *et al*. 2006; Hector *et al*. 2010; Hautier *et al*. 2014), yet the last decade of research has shown that the dynamics of metacommunities often differ from those of the local communities of which they are composed (Leibold *et al*. 2004; Laliberté *et al*. 2013). Quantifying the processes that determine the stability of ecosystem functioning at scales beyond the traditional ecological study plot is a critical step toward predicting the consequences of environmental change (e.g. biodiversity loss, spatial homogenisation) at spatial scales relevant for land management.

**Figure 1:**
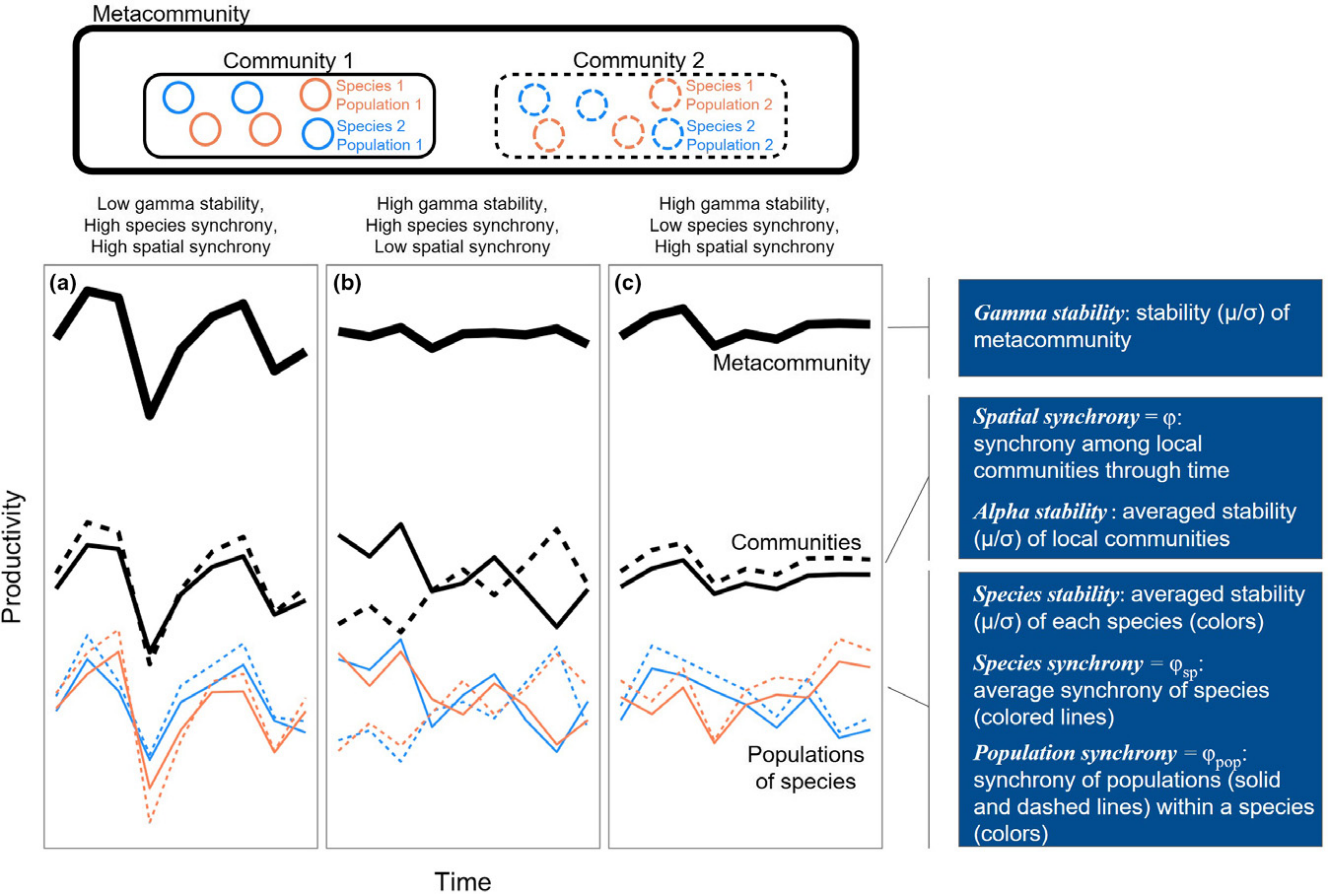
Conceptual figure showing how stability and synchrony at various spatial scales within a metacommunity combine to determine the stability of ecosystem function (here, productivity). In (a), high synchrony of species within and among local communities results in low stability at the scale of the metacommunity. In (b), species remain synchronised within local communities, but the two communities exhibit asynchronous dynamics due to low population synchrony among local patches. This results in relatively high gamma stability. Lastly, in (c), species exhibit asynchronous dynamics within local communities through time, and species‐level dynamics are similar across communities (i.e. high population synchrony). This results in relatively high gamma stability. Blue boxes on the right outline stability components and mechanisms, and the hierarchical level at which they operate. Adapted from Mellin *et al*. (2014).

Although the term stability has a range of different meanings in ecology (Grimm & Wissel 1997), we define stability here as the mean of an ecosystem function, such as net primary productivity, divided by its temporal standard deviation (Tilman 1999). In a MacArthur lecture, Levin (1992) formalised the idea that the stability of ecosystem processes through space or time is increased as the spatial scale of focus increases. Additionally, Levin stressed the importance and the need for detailed understanding of how and why stability changes across scales. More recently, Gurevitch *et al*. (2016) discussed the usefulness of aggregating population dynamics to inform processes occurring at larger spatial scales, and recent theoretical work has developed a hierarchical framework that can help integrate processes that affect stability from the local to the landscape or metacommunity scale (Fig. 1; Mellin *et al*. 2014; Wang & Loreau 2014). This framework provides quantifiable terms that represent stability and synchrony at various spatial scales.

For clarity, and to be consistent with recent theoretical work (Wang & Loreau 2014, 2016), we use the following terminology to refer to and integrate across three levels of ecological organisation: (1) *populations* of different *species* combine to create (2) local *communities*, which aggregate to form (3) *metacommunities*. At any given scale, the stability of ecosystem functioning is determined by the stability of its component parts (e.g. populations or species at the community level; communities at the metacommunity level) and how synchronous the component parts are from year to year (Fig. 1; Loreau & de Mazancourt 2013; Hautier *et al*. 2014; Hallett *et al*. 2014). Synchrony measures the degree to which component parts respond similarly to environmental fluctuations, interannual competitive effects, and demographic stochasticity (Loreau & de Mazancourt 2008; Tredennick *et al*. 2017). For example, if two species have very dissimilar responses to environmental fluctuations, then synchrony will be low. The same is true for communities – if two local communities have differential responses to environmental fluctuations, perhaps due to different species composition, then the synchrony of the two communities will be low (Wang & Loreau 2016).

As an example of the framework outlined above, first consider a perfectly homogeneous and deterministic landscape: the average stability of all species (*species stability*) and the degree to which they are synchronised (*species synchrony*) jointly determine the stability of local community function (*alpha stability*). In this homogenous landscape, all communities respond similarly through time, and thus alpha stability directly scales up to determine the stability of the metacommunity (*gamma stability*; Fig. 1a,c black lines). In such a homogenous system, the identity and stability of dominant species (Smith & Knapp 2003; Polley *et al*. 2007; Loreau & de Mazancourt 2013), species richness and diversity (Tilman 1996; Naeem & Li 1997; Yachi & Loreau 1999; Tilman *et al*. 2006; Gross *et al*. 2014; Isbell *et al*. 2015), and functional diversity (Bai *et al*. 2004; Polley *et al*. 2013) within local communities are important drivers of gamma stability.

In real landscapes, which are neither completely homogenous nor perfectly deterministic, different local communities will vary in their response to interannual fluctuations in environmental drivers, such as weather or disturbance, or due to demographic stochasticity (Gurevitch *et al*. 2016). How much these local communities vary with respect to one another determines the level of synchrony of ecosystem functioning at the metacommunity scale. Just as low species synchrony can stabilise a local community (Loreau & de Mazancourt 2008, 2013), low synchrony amongst local communities (*spatial synchrony*) can increase gamma stability, even if alpha stability is low (Fig. 1b). This pattern could arise through species turnover resulting in different communities existing across space (beta diversity), or by variations in local microhabitat across local communities (e.g. heterogeneity of soil fertility) moderating population and/or species responses through time (Doak & Morris 2010). There is compelling theoretical justification for how stability may be regulated by spatial synchrony at larger spatial scales (Turner *et al*. 1993; Wang & Loreau 2014, 2016), but quantification of the stabilising role of spatial synchrony or its association with biodiversity is scarce (McGranahan *et al*. 2016).

Here, we use an intercontinental database of plant species abundance information to quantitatively partition gamma stability into its hierarchical components (Fig. 1; Wang & Loreau 2014). We addressed two major questions. First, relative to local‐scale stability, how important is spatial synchrony for regulating stability at larger spatial scales? Second, what are the major ecological predictors of synchrony and stability within and across metacommunities? Regarding the second question, we tested two sets of non‐exclusive hypotheses: (H1a) spatial synchrony across metacommunities is negatively related to species turnover among local communities (e.g. beta diversity). This could be driven by unique responses of different local communities to interannual environmental variation; alternately, (H1b) spatial synchrony across metacommunities is positively related to synchrony among species populations. This could result from spatial variation of physical characteristics or competition among local communities or demographic stochasticity. (H2) Alpha diversity will be positively related to alpha stability and negatively related to species synchrony. We hypothesised this because communities with higher alpha diversity often have higher primary productivity as well as greater variety of plant growth strategies. In summary, we seek to quantify the determinants of ecosystem stability at the metacommunity scale, and to understand the ecological factors that contribute to stability across spatial scales and levels of organisation.

## Materials and Methods

### Data sets

Disentangling the drivers of gamma stability requires estimates of species abundance and/or primary productivity through time at the metacommunity, community and species/population levels (Fig. 1). In February of 2016, we searched the CoRRE (http://corredata.weebly.com/) database to obtain species‐level abundance data from 62 herbaceous community studies distributed around the globe (Table S1 and Fig. S1). We extracted data collected in control (no manipulation) plots from data sets that met the following criteria:

1. Contained at least three replicate plots in all years.
2. Contained at least 3 years of measurements for all replicate plots.
3. Plot sizes consistent in all replicates.
4. Starting community intact and not undergoing strong primary or secondary succession (i.e. due to plowing, weeding, seeding).
5. Included absolute species‐level abundance.
6. Nutrients not added before or during the measurement period.

The data represent ecosystems spanning climatic gradients of mean annual precipitation from 168 to 1400 mm (mean: 725 mm; median: 751 mm) and mean annual temperature from −12 to 22 °C (mean: 7.0 °C; median: 8.5 °C). Mean aboveground net primary productivity (ANPP) among studies varied from 1.5 to 1002 g m^−2^ (mean: 311.6 g m^−2^; median: 222.1 g m^−2^), and study‐wide richness varied from 5 to 140 species (mean: 59 species; median: 55 species). The number of replicates sampled varied from 3 to 18 (mean: 6.2; median: 5), experimental length varied from 3 to 30 years (mean: 7.6 years; median: 5 years), and replicate sizes ranged from 0.03 to 10 m^2^ (mean: 1.4 m^2^; median: 0.6 m^2^). See Table S1 for more information about included data sets.

Each study included several replicate plots that were relatively close in space (Table S1). We treated these plots as individual communities, and collections of plots as metacommunities. When groups of plots within a study were in different study sites (e.g. different watersheds, fields), they were separated and treated as distinct metacommunities. Absolute percent abundance of species within plots were measured either by visual estimates of percent cover (*N* = 35), presence/absence (*N* = 2), line‐intercept (*N* = 1), biomass (*N* = 12), primary productivity (*N *= 6), pin hits (*N* = 3), or stem density (*N* = 3). Plot‐level ANPP was estimated in or next to the same plots as species percent abundances in 35 of the metacommunities via clipping (*N* = 26), pin hit/point intercept (*N* = 5), allometric calibrations (*N* = 3), or percent cover calibrations (*N* = 1).

### Calculating stability components

Within each study site (treated here as a metacommunity), we used species abundance and plot‐level ANPP data to calculate alpha stability, spatial synchrony, and gamma stability following Wang & Loreau (2014, 2016) (Table 1). For clarity purposes, we inversed the *coefficient of variability* terms presented in Wang & Loreau (2014, 2016) to represent *stability*. Gamma stability (γ_*stb*_) is a measure of the temporal variability of ecosystem functioning of the entire metacommunity, calculated as follows:

(1) $$\gamma_{stb}=\frac{\mu_{M}}{\sigma_{M}}$$

where σ_*M*_ is the temporal standard deviation and μ_*M*_ is the temporal mean of summed total abundance in metacommunity *M*. For species cover data sets, we used the sum of total cover across all plots as total metacommunity abundance; for ANPP data sets, we used summed ANPP across all plots.

**Table 1:** Summary of variability metrics, notation, and descriptions

Name	Notation	Technical description	Ecological description
Gamma stability	γ_*stb*_	Temporal stability of abundance of all plots within a study site	Ecosystem stability of a collection of local communities – at the metacommunity scale
Spatial synchrony	φ	The degree that plot‐level abundances align to one another through time within a study site	The level to which local communities vary similarly in certain years. Potentially influenced by heterogeneity of communities, populations, and/or physical conditions across communities
Spatial stabilisation	$\sqrt{\frac{1}{\varphi }}$	The inverse of the among‐plot synchrony of local community abundance through time	The factor by which temporal stability is increased when moving from the community to the metacommunity scale
Alpha stability	α_*stb*_	Temporal variability of total plant abundance at the plot scale	Stability at the local community scale. Influenced by growth strategies of component species (*Sp*_*stb*_) and/or by the synchrony of different species abundance through time (φ_*sp*_)
Species stability	*Sp*_*stb*_	Species‐level stability within a plot, first averaged over species within plots, then averaged over plots to obtain a single value for each study site	How variable individual species abundances are from year to year. Heavily influenced by growth strategies of dominant species
Species synchrony	φ_*sp*_	The degree that species abundances align with other species’ abundance through time, averaged over plots to obtain a single value for each study site	Different species responding in different ways through time. Likely driven strongly by functional diversity among species within a community
Population synchrony	φ_*pop*_	The degree that a species’ abundance through time within one plot aligns with its abundance in different plots. Averaged across species to obtain a single value for each study site	Different populations of the same species responding differently through time in different communities. Likely driven by genotypic heterogeneity, interspecific competition, and/or different physical environments among patches

Note

See Wang & Loreau (2014) for more detail about calculating variability metrics.

Alpha stability (α_*stb*_) is a measure of the average temporal stability of plant abundance or productivity at the local community scale. We obtained alpha stability for each site by calculating the temporal coefficient of variation of total community abundance (summed species cover or plot‐level ANPP) for each local community (plot), weighting by local community abundance, and taking the inverse:

(2) $$\alpha_{stb}={\left(\sum_{i}\frac{\mu_{i}}{\mu_{M}}\times \frac{\sigma_{i}}{\mu_{i}}\right)}^{-1}$$

where μ_*i*_ is the temporal mean of total abundance in community *i*, μ_*M*_ is the temporal mean of total abundance in metacommunity *M*, and σ_*i*_ is the temporal standard deviation of total abundance in community *i*. As above, for total abundance we used total summed absolute species cover or ANPP for species cover and ANPP data sets, respectively.

Spatial synchrony (φ) represents the similarity of temporal fluctuations of different communities (within a metacommunity), and is calculated as:

(3) $$\varphi =\frac{\sum_{i,j}w_{ij}}{{\left(\sum_{i}\sqrt{w_{ii}}\right)}^{2}}$$

where *w*_*ij*_ is the temporal covariance between communities *i* and *j*, and *w*_*ii*_ is the temporal variance of community *i*, as referenced from a covariance matrix. In addition to synchrony of local community abundance through time, spatial synchrony can also incorporate spatial unevenness if mean and variance of plot abundance is not equal among plots (Wang & Loreau 2014). We do not think spatial unevenness was a large component of spatial synchrony here because we did not find obvious and consistent violation of the assumption of equal means and variance of abundance across plots (Fig. S2).

Lastly, we used the reciprocal of spatial synchrony to represent the degree to which squared stability is increased due to spatial dynamics (eqn (6) in Wang & Loreau 2014). So, we term *spatial stabilisation* as the square root of 1/ϕ, which corresponds to the amount of stability enhanced when moving from the community to metacommunity level:

(4) $$\sqrt{\frac{1}{\varphi }}=\frac{\gamma_{stb}}{\alpha_{stb}}$$

To test for potential bias of plot size on the spatial stabilisation metric, we calculated spatial stabilisation, using the entire database and a subset of metacommunities containing 1 m^2^ plots to assess the range, mean, and median spatial stabilisation values across our database. We present the summary statistics of spatial stabilisation across the entire database because these values were qualitatively similar when using only metacommunities with 1 m^2^ plots (Table S2).

We used species abundance data to calculate two temporal metrics at the species level and one at the population level – species synchrony, species stability, and population synchrony. Plot‐level ANPP data were mostly not parsed by species, so we were unable to calculate species‐level synchrony or stability for ANPP. We calculated species synchrony (φ_*sp*_) within each community following Loreau & de Mazancourt (2008):

(5) $$\varphi_{sp,i}=\frac{\sum_{k,l}w_{kl,i}}{{\left(\sum_{k}\sqrt{w_{kk,i}}\right)}^{2}}$$

where *w* is the temporal covariance matrix comparing abundances of species *k* and *l* within community *i*. Species synchrony values were averaged across plots, weighting by total plot abundance, to obtain a single value for each metacommunity. There is a mathematical bias between species richness and the species synchrony metric in eqn (5), so for all analyses which compared species synchrony with measures that incorporate information about species richness (e.g. Simpson's diversity), we also ran the analyses using a species synchrony metric that is not biased by species richness (Gross *et al*. 2014). All such analyses yielded very similar results to those obtained using eqn (5), so we report results using the species synchrony metric in eqn (5). Species stability (*Sp*_*stb*_) for each community was calculated as:

(6) $$Sp_{stb,i}={\left(\sum_{j}\frac{\mu_{j(i)}}{\mu_{i}}\times \frac{\sigma_{j(i)}}{\mu_{j(i)}}\right)}^{-1}$$

where μ_*j*(*i*)_ is the mean of species *j*'s abundance through time in community *i*, μ_*i*_ is mean total abundance in community *i*, μ_*j*(*i*)_ is the mean abundance of species *j* in community *i*, and σ_*j*(*i*)_ is the temporal standard deviation of species *j* in community *i*. As with species synchrony, we averaged species stability across communities, weighting by each species’ relative abundance and then by total plot abundance to obtain estimates of *Sp*_*stb*_ for each metacommunity. Finally, for each species, we calculated population synchrony as follows:

(7) $$\varphi_{pop,i}=\frac{\sum_{m,n}w_{mn}}{{\left(\sum_{m}\sqrt{w_{mm}}\right)}^{2}}$$

where for each species *i* present within a metacommunity, *w*_*mn*_ is the temporal covariance between populations (species abundance in single plots) *m* and *n*, and *w*_*mm*_ is the temporal variance of population *m*, as referenced from a covariance matrix. We then averaged across species, weighting by species’ relative abundance, to obtain a single population synchrony value for each metacommunity. See Table 1 for a summary of stability metrics and components. We calculated all metrics in R (R Core Team 2016), and used the ‘synchrony()’ function (‘codyn’ package, Hallett *et al*. 2016) to calculate synchrony metrics.

### Calculating biodiversity

We calculated alpha and beta diversity indices to compare with gamma stability, alpha stability, and spatial synchrony. To estimate alpha diversity, we calculated the Simpson's index (*D*):

(8) $$D=\sum_{i=1}^{S}p_{i}^{2}$$

where *p*_*i*_ is the relative abundance of species *i* and *S* is the number of species within a plot. We also used Shannon–Wiener index (*H′*):

(9) $$H^{\prime }=-\sum_{i=1}^{S}p_{i}\times \ln\left(p_{i}\right)$$

We calculated these for each plot in each year and took the average. These metrics incorporate information about species richness and abundance. We also calculated diversity of entire metacommunities by averaging cover values across plots and applying eqns (8) and (9).

Beta diversity, or compositional dissimilarity among local communities, was calculated for each metacommunity using multivariate dispersion techniques with species abundance data (Anderson *et al*. 2006), which estimates the distance of each community to the metacommunity centroid in multivariate space. Distances were averaged across communities and years to estimate beta‐diversity for a metacommunity. Beta‐diversity, Simpson's, Shannon‐Wiener indexes were calculated using the ‘betadisper()’ and ‘diversity()’ functions in R (‘vegan’ package, Oksanen *et al*. 2016).

### Quantifying components of gamma stability

A major goal was to attribute variation in gamma stability among metacommunities to specific lower level attributes like alpha stability and spatial synchrony. We proceeded in three steps: (1) we partitioned the variation of gamma stability among alpha stability and spatial synchrony; (2) we partitioned the variation of gamma stability among species synchrony, species stability, and spatial synchrony; and (3) we conducted bivariate linear regressions among stability‐driver pairs to further assess and visualise the influence of lower‐level processes on higher‐level variability. Few studies measured ANPP information at the species‐level, so we were limited to steps 1 and 3 for ANPP. Plot size varied across many studies used in our analyses, likely due to investigator knowledge of the different spatial scales of ecological processes across sites. We chose to trust principle investigator decisions on appropriate plot sizes used in individual studies, but we also found qualitatively similar results when we performed our variance partitioning analyses using only studies having 1 m^2^ plots (these were the most common; Fig. S3). Additionally, we performed linear regressions between spatial and population synchrony and study duration, plot number, plot size, and metacommunity size to determine whether underlying methodological factors might be driving relationships between stability components.

Linear regressions were used to compare alpha and beta diversity metrics with gamma stability, alpha stability, spatial synchrony, species stability, and species synchrony across sites. This allowed us to determine whether (1) diversity metrics in isolation were associated with local stability and synchrony dynamics, and (2) whether these impacts scaled up to affect larger (gamma) spatial scales. To account for cross‐site patterns of potentially confounding factors, such as environmental variability or disturbance regime, we conducted regressions comparing Simpson's diversity with alpha stability and species synchrony across plots within sites. For this, we only incorporated sites having five or more plots (44 metacommunities) to avoid biasing results by including studies with small sample size. Population synchrony was compared with spatial synchrony also using linear regression. We used the ‘varpart()’ function in R (‘vegan’ package, Oksanen *et al*. 2016) to conduct variance partitioning and the ‘lm()’ function in R to fit linear regressions. The variance partitioning analysis provides variance explained by each metric alone and shared. We used log‐transformed metrics for all statistical analysis to account for non‐normality. R code and derived data for all analyses have been deposited on Figshare (https://doi.org/10.6084/m9.figshare.5384167). See http://corredata.weebly.com/ for inquiries concerning raw data.

## Results

### Quantifying influence of stability components

**Figure 2:**
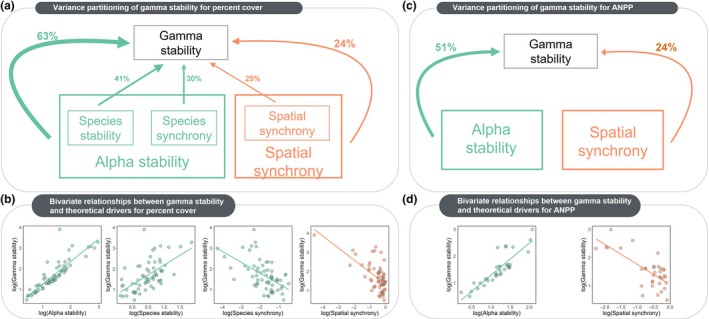
Variance partitioning of gamma stability among theoretical components (a,c) and independent relationships between predictors and metacommunity stability (b,d) for percent cover (a,b) and ANPP data (c,d). In (a), we use curved lines to represent the partitioning of variance among alpha stability and spatial synchrony, and straight lines to represent the finer‐scale partitioning of variance among species stability, species synchrony, and spatial synchrony. Note that species stability and species synchrony determine alpha stability. All theoretical drivers are significant independent predictors of gamma stability (slopes of regression lines in panels (b) and (d) all *P *<* *0.0001). For variance partitioning and independent regressions, theoretical drivers are log‐transformed to match theoretical predictions (Wang & Loreau 2014).

Using species abundance data, species stability, species synchrony, and spatial synchrony contributed 41, 30, and 25%, respectively, to explaining the cross‐site variance of gamma stability (Fig. 2a). Alpha stability, which represents the combined effects of species stability and synchrony, alone explained 63% of the variance in gamma stability among sites, while spatial synchrony alone explained 24% (Fig. 2a). The remaining 13% of the variance in gamma stability was explained by covariance between the two predictors. All individual drivers (alpha stability, spatial synchrony, species stability, and species synchrony) were significant (*P *<* *0.01) predictors of variation in gamma stability among sites (Fig. 2b). To ensure the spatial synchrony‐gamma stability relationship was not being driven by one site that had very low spatial synchrony (Fig. 2b – far right panel), we reran the analysis with the data point removed. The relationship was still significant and the variance partitioning results were qualitatively similar (Fig. S4).

Results using ANPP data yielded similar findings: alpha stability alone explained 51% and spatial synchrony alone explained 24% of the variation in gamma stability (Fig. 2c). Jointly, alpha stability and spatial synchrony explained the additional 25% of the variation. For ANPP, both alpha stability and spatial synchrony were again significant (*P *<* *0.01) independent predictors of variation in gamma stability among sites (Fig. 2d).

**Figure 3:**
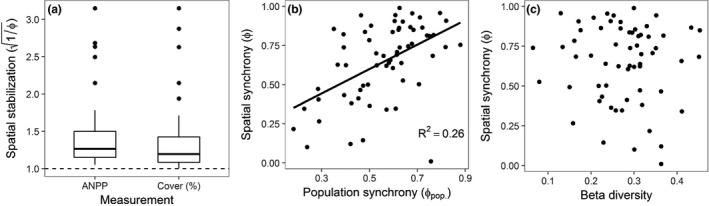
(a) Boxplots of site‐level spatial stabilisation (square root of the inverse of spatial synchrony) from ANPP and percent cover data. Spatial stabilisation measures how much stability is increased from alpha (local community) to gamma (metacommunity) spatial scales. A value of 1 (dashed horizontal line) indicates no increase. (b) Cross‐site relationship between population synchrony (averaged across species for each metacommunity) and spatial synchrony (c) Cross‐site relationship between spatial turnover of species (i.e. beta diversity calculated using multivariate permutational dispersion techniques) and spatial synchrony. Relationships without trend‐lines represent non‐significant relationships at α = 0.05. We did not log‐transform metrics for these regressions because doing so did not improve normality.

The spatial stabilisation factor provides information about how much stability is increased when moving from smaller to larger spatial scales. Using both species percent abundance and ANPP data, we found a wide range of spatial stabilisation factors across metacommunities, ranging from 1.01 to 3.15 (mean = 1.38, median = 1.21). This indicates that spatial synchrony increased stability from the community to metacommunity scale by as little as 1% in some metacommunities and as much as 315% in others (Fig. 3a). We found significantly positive cross‐site relationships between the size of a metacommunity and its spatial stabilisation, using ANPP data (*F*_2,52_ = 8.7, *P* < 0.01, *R* ^2^ = 0.26; Fig. S5) and species cover data (*F*_2,27_ = 4.0, *P* = 0.05, *R* ^2^ = 0.07).

### Predictors of spatial synchrony

We did not find a significant relationship between beta diversity and spatial synchrony using species abundance (*F*_1,60_ = 0.11, *P* = 0.74; Fig. 3c) or ANPP data (*F*_1,31_ = 2.09, *P* = 0.16). Alternately, we found spatial synchrony and population synchrony (synchrony of same‐species abundances across local communities) were positively correlated across sites (*F*_1,60_ = 15.02, *P* < 0.01; Fig. 3b). To check whether the relationship between population and spatial synchrony was driven by simultaneous relationships of spatial and population synchrony with methodological variables, we regressed the synchrony metrics with the number of plots in a metacommunity, the size of plots in a metacommunity, and the duration of the study. We found no significant regressions between spatial or population synchrony and any of these methodological factors (Table S3).

### Species diversity as a predictor of stability and spatial synchrony

**Figure 4:**
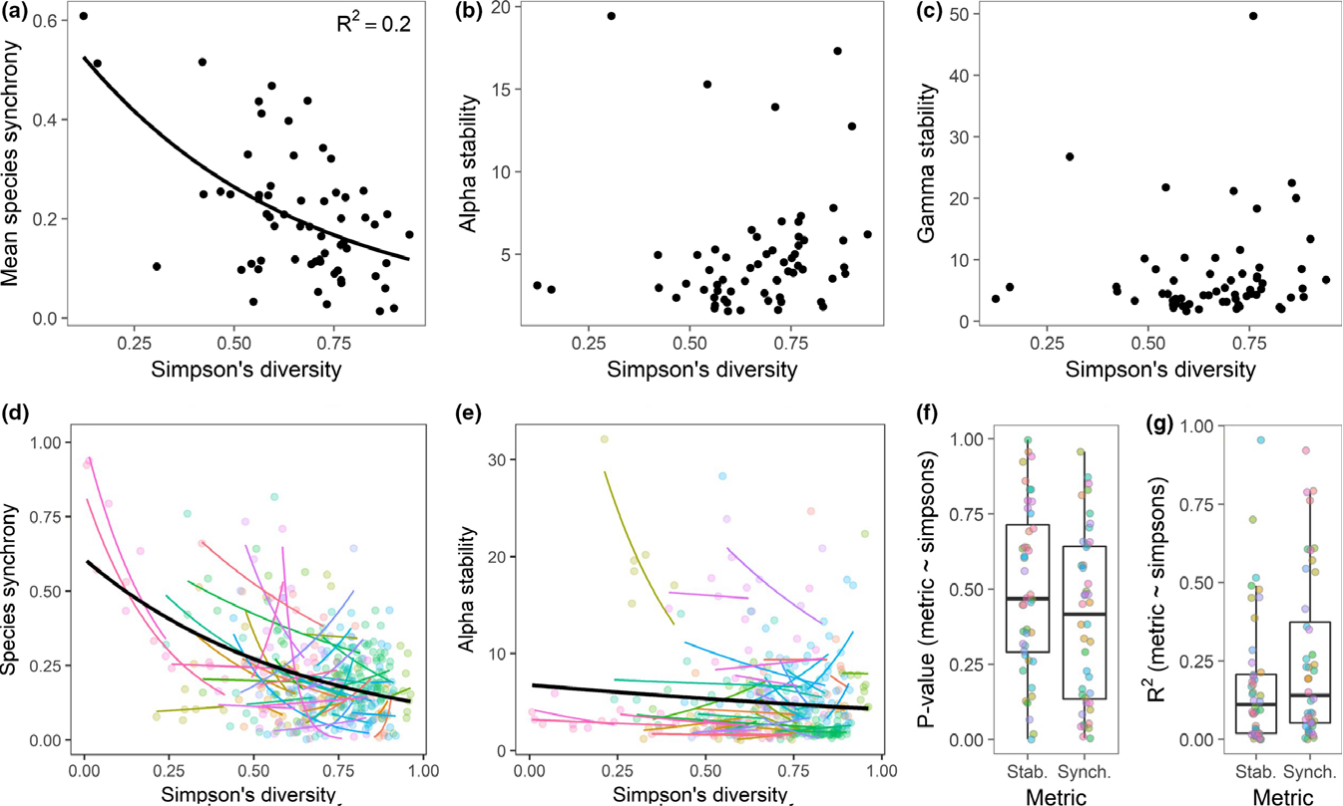
Bivariate relationships between Simpson's diversity within each meta‐community, and (a) species synchrony, (b) alpha stability, and (c) gamma stability. In panels (a–c), Simpson's diversity was calculated using species level data within plots, then averaged for each site. Relationships in a‐c without trend lines are non‐significant at α = 0.05. In panels (d–g), Simpson's diversity, species synchrony, and alpha stability was calculated for each plot within a metacommunity. Regressions comparing Simpson's diversity versus species synchrony (d) and alpha stability (e) were conducted separately for each metacommunity. Different colors indicate different sites and thick black lines represent overall trends. Panels (f) and (g) summarise *P*‐values and *R* ^2^ values for individual metacommunity regressions.

We first looked at local stability mechanisms that we predicted would be impacted by alpha diversity: species synchrony and alpha stability. We found species synchrony was negatively related to alpha diversity across sites (*F*_1,60_ = 14.7, *P* < 0.01; Fig. 4a), but did not find significant relationships between alpha diversity and alpha stability (*F*_1,60_ = 2.30, *P* = 0.13; Fig. 4b) or gamma stability across sites (*F*_1,60_ = 0.78, *P* = 0.38; Fig. 4c). When looking across plots within metacommunities having at least five plots, we found only two of 44 sites with significant alpha diversity‐alpha stability relationships at α = 0.05, and only six sites with significant alpha diversity‐species synchrony relationships (Fig. 4d–g). We conducted these same analyses, using Shannon's diversity, with qualitatively similar findings (Table S4). We found similar results when comparing species diversity of the entire metacommunity with stability and synchrony metrics (Fig. S6; Table S5).

## Discussion

Ecosystem stability provides information about the predictability and consistency of ecosystem functioning through time. We used an intercontinental database of herbaceous plant species abundance and ANPP to conduct the first broad empirical test of recent theory (Wang & Loreau 2014) by (1) quantifying the relative contributions of alpha stability and spatial synchrony on gamma stability, and (2) searching for predictors of stability and synchrony at multiple spatial scales. Across the 62 metacommunities we studied, we found that stability and synchrony at the local community and species level were important components of stability at larger spatial scales (Fig. 2). When moving from the species to the local community scale, and from the local community to the metacommunity scale, stability increased due to asynchrony among species and local communities. We found synchrony among local populations residing in different local communities was an important predictor of spatial synchrony. Below, we go into the implications of these findings in more depth.

### Asynchrony enhances stability at larger spatial scales

We found much of the variance in cross‐site patterns of metacommunity stability was explained by alpha stability (Fig. 2), and that this was driven by both species stability and the degree of species synchrony in local communities. This result coincides with many studies showing that ecosystem stability can be impacted by local species dynamics, such as the reordering of dominant species under altered environmental conditions (Smith & Knapp 2003; Grman *et al*. 2010; Cavin *et al*. 2013; Avolio *et al*. 2014; Koerner *et al*. 2016; Wilcox *et al*. 2016), invasive species altering interspecific competition dynamics (Melbourne *et al*. 2007; Ricciardi *et al*. 2013), and/or shifts in functional composition and diversity occurring under chronic climatic shifts (Hoover *et al*. 2014). Additional factors that might impact alpha and gamma stability are altered disturbance regimes (White & Jentsch 2001) and ecosystem state transitions (Potts *et al*. 2006). These community and ecosystem dynamics can impact the temporal stability of local ecosystem function, which, as we show, scales up to impact stability at larger spatial scales (Fig. 2).

Spatial stabilisation (here, directly derived from spatial synchrony) of ecosystem dynamics through time enhanced the temporal stability transferred from community to metacommunity scales by factors ranging from as little as 1.01 to 3.15 (Fig. 3a). This means that asynchrony among local communities enhanced metacommunity stability by a range of 1 to 315%. Although our results are unable to provide a generalised number for the stabilising force of spatial synchrony, because it inherently increases with spatial scale, our results do show that even with relatively small increases in spatial scale, asynchrony among local communities is an important driver of large‐scale stability. This is especially remarkable because most data sets used in our analysis describe local communities that are relatively close together in space and were often chosen to be similar to one another. Thus, our results likely represent a conservative estimate of the importance of spatial synchrony. Indeed, we found positive correlations between the size of a metacommunity and its spatial stabilisation factor (Fig. S5), corresponding to recent work by Wang *et al*. (2017). Thus, over larger spatial extents, where the intrinsic heterogeneity and species turnover should be greater, the importance of spatial synchrony to ecosystem functioning should also be greater (Wang & Loreau 2014; McGranahan *et al*. 2016).

There are two naive expectations for the magnitude of spatial stabilisation. First, we might expect no stabilisation if all communities fluctuate in perfect synchrony through time. Second, we might expect stabilisation to be directly proportional to the number of local communities if all communities fluctuate independently (Wang & Loreau 2014). In reality, as we have found here, spatial stabilisation will fall somewhere between these theoretical bounds. We found spatial stabilisation was often far from one, meaning that local communities were not perfectly correlated through time. We also found that spatial stabilisation was often less than the number of plots sampled (Fig. S7), meaning that local communities were positively, though not perfectly, correlated through time. Species turnover in space or unique responses of within‐species populations among plots could cause less than perfect spatial synchrony. We examine each in turn.

First, spatial turnover of community composition (i.e. species identities and abundances) can result in functional differences among communities, and reduce synchrony of local community functioning in particular years (Mellin *et al*. 2014). Second, even when species composition is similar, the functioning of species across communities may exhibit different temporal dynamics due to various factors, including differences in local environmental characteristics (Ladwig *et al*. 2012) or demographic stochasticity. For example, local populations of species existing in deeper, wetter soils may be less responsive during low rainfall years than local populations in shallower soils (Heisler & Knapp 2008).

Contrary to our expectations, we found that beta diversity (an estimate of spatial turnover of species; Anderson *et al*. 2006; Avolio *et al*. 2015) was a poor predictor of spatial synchrony (Fig. 3c). We think this likely stems from the fact that beta diversity, as we calculated it, does not consider the functional similarity of different species, and many of the plots (which we treat as local communities) within a metacommunity were chosen to be functionally similar. We suggest that functional differences among local communities may be a better predictor of spatial synchrony (McGill *et al*. 2006; Polley *et al*. 2013), and incorporating species functional information into measures of beta diversity (e.g. species traits; Lavorel & Garnier 2002; Zhang *et al*. 2012) will likely yield more insight into the biotic drivers of spatial synchrony.

We found that many species’ populations fluctuated asynchronously among communities (Fig. S8). We calculated an average population synchrony for each metacommunity (eqn (7)), and this explained 26% of the variation in spatial synchrony across sites (Fig. 3b). This is not particularly surprising in the absence of strong beta diversity‐synchrony relationships because these are both measures of synchrony dynamics across space. Consequently, we suggest asynchrony among populations has the potential to increase gamma stability. Asynchronous population responses could be driven by multiple factors, such as heterogeneity of physical characteristics among patches, genotypic or phenotypic differences among populations residing in different communities (Avolio *et al*. 2013; Chang & Smith 2014), different species interactions occurring in different communities (Tilman *et al*. 2006), spatio‐temporal patterns of successional dynamics, and demographic stochasticity. We posit that population‐specific responses drive patterns of spatial synchrony jointly with spatial turnover of functionally different communities, especially at even larger spatial scales (Doak & Morris 2010). A future challenge is to quantify the contribution of different mechanisms underlying population synchrony, similar to recent work on species synchrony (Tredennick *et al*. 2017).

### Alpha diversity versus stability and synchrony

Based on previous experimental evidence (Tilman *et al*. 2006; Hector *et al*. 2010), we predicted that the diversity of local communities (alpha diversity) would be correlated with species synchrony, alpha stability, and ultimately gamma stability. A major mechanism by which local species diversity can increase stability of local communities is by reducing the synchrony of species’ abundances through time (Loreau & de Mazancourt 2008; Isbell *et al*. 2009). Indeed, we found evidence for this mechanism as species synchrony was generally lower in metacommunities having higher alpha diversity (Fig. 4a). However, we did not find that this scaled up to impact alpha or gamma stability across sites (Fig. 4b–c). This corresponds with previous theoretical work suggesting that the effect of alpha diversity on gamma stability may be small compared to other biotic and abiotic forces (Wang & Loreau 2016). Thus, it may be difficult to detect diversity effects on stability in short and noisy time series of community dynamics where cross‐site patterns of interannual variation in weather and disturbance levels (e.g. herbivore pressure) may also have strong impacts on stability.

Additionally, we found few significant alpha diversity‐alpha stability relationships across local communities within sites (Fig. 4d–g). This may be due to different relationships between alpha diversity and stability in different local communities. For example, in some local communities, greater species diversity might enhance species stability by increasing productivity (the numerator in the stability formula; de Mazancourt *et al*. 2013). However, in other local communities, greater species richness may decrease species stability if higher diversity leads to more competitive interactions and larger population fluctuations (Loreau & de Mazancourt 2013). So, although we think that changes in biodiversity through time can impact gamma stability though increases in species synchrony, other site‐level factors may cloud the apparentness of such an effect.

## Conclusions

Ecosystem stability provides actionable information about the predictability and persistence of multiple ecosystem services. Understanding stability at ecosystem or metacommunity scales requires knowledge of how stability is maintained when moving from smaller to larger spatial scales. Yet, most of our knowledge about the drivers of ecosystem stability comes from relatively small‐scale studies. Consistent with emerging theory (Wang & Loreau 2014), we found that the stability of spatially larger systems results both from the stability of local communities within the larger system, and from the degree of synchrony among these local communities. Contrary to expectations, beta diversity was not predictive of functional differences among local communities within a metacommunity. Instead, it appears that population‐specific responses to environmental conditions/disturbance regimes or different species interactions across local communities may play a large role in promoting stability at larger spatial scales. Based on these findings, we suggest that homogenisation of physical characteristics and/or plant populations across space may substantially reduce ecosystem stability.

## Author Contributions

KRW and ATT designed and led the manuscript; KRW, ATT, SEK and EG participated in original idea generation; MLA and KJL led the collection of the database with the help of KRW, SEK, EG, GRH, FI, and DSJ; MLA, KJL, GRH, FI, DSJ, JMA, AHB, EB, EHB, WDB, AB, JFC, SLC, GD, AE, LG, AJ, CK, KK, AKK, JK, YL, JRM, PM, VO, JP, JP, CHR, OS, MDS, NAS, LS, DT, SRW, ZX, LY, YQ and YZ organised and collected abundance and productivity data at various sites, data analysis was done by KRW, ATT, SEK, EG and LMH; all authors were involved in the writing and editing process.

## Data Accessibility Statement

R code and derived data for all analyses are available on Figshare (https://doi.org/10.6084/m9.figshare.5384167). See http://corredata.weebly.com/ for inquiries concerning raw data.
