# Supplementary material for: Asynchrony among local communities stabilises ecosystem function of metacommunities
Source: Ecol Lett. 2017 Oct 24;20(12):1534–45. doi: 10.1111/ele.12861 (PMC6849522; doi:10.1111/ele.12861)
Supplement: Supplementary file 1 [file ELE-20-1534-s001.docx]

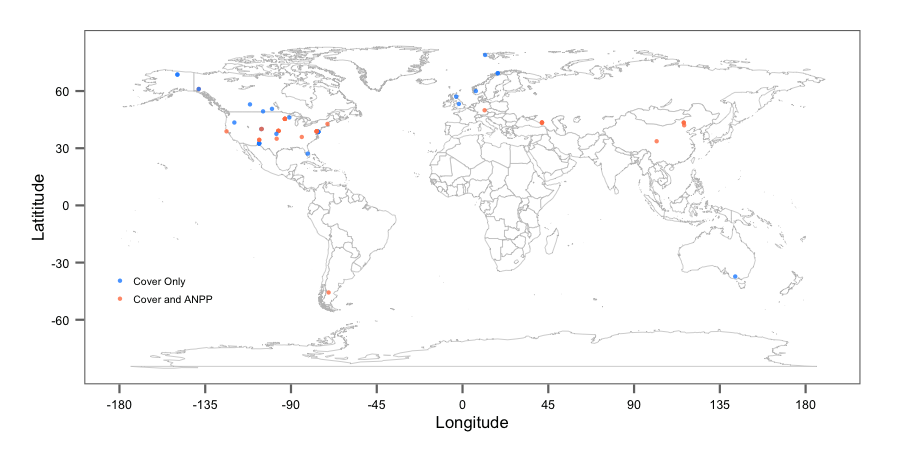


Figure S1. Map showing data-sites having either plant species abundance alone (blue), or both plant species abundance and primary production (orange) information. Data were obtained from control plots of experiments in herbaceous vegetation systems.


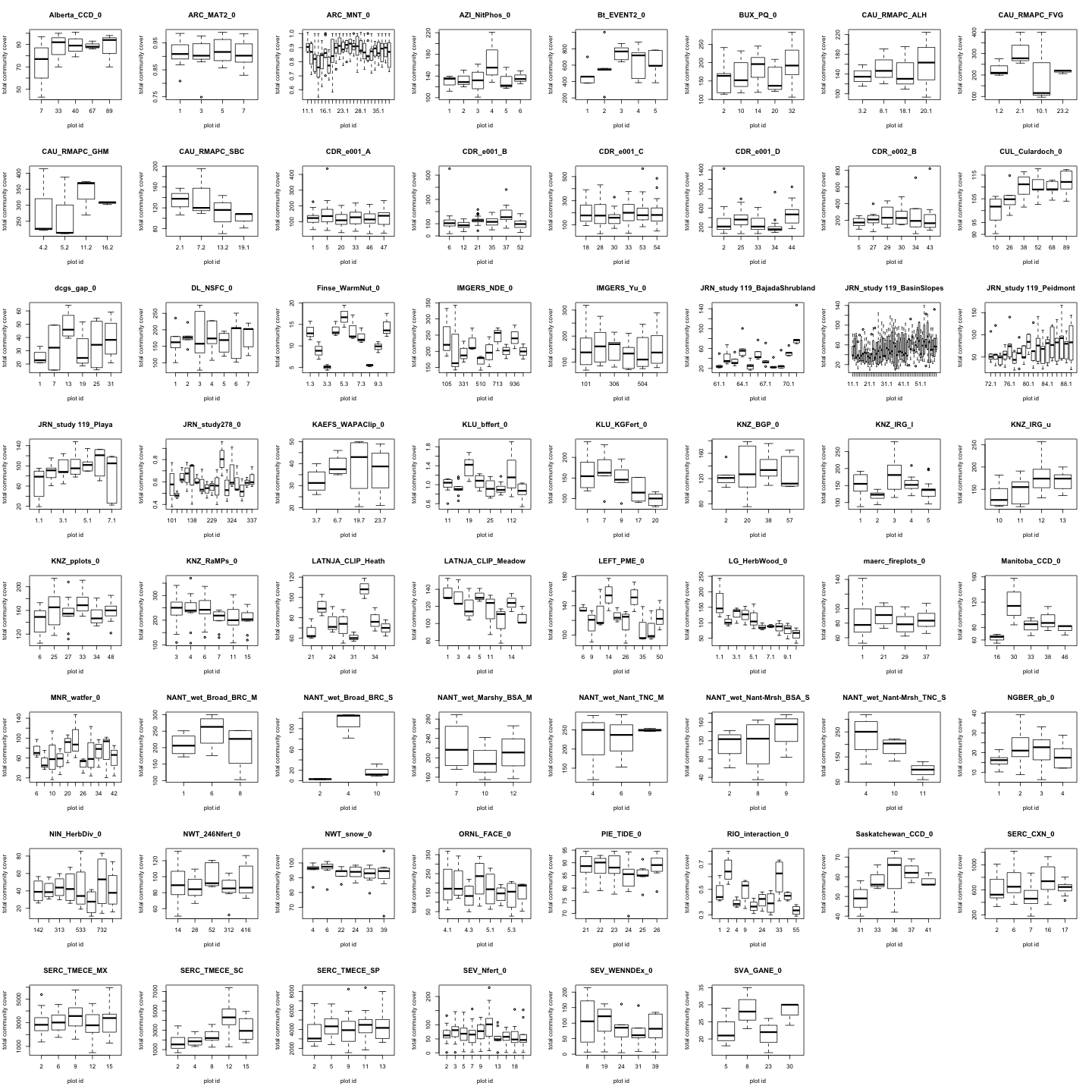


Figure S2. Boxplots of plot-level vegetation abundance through time.


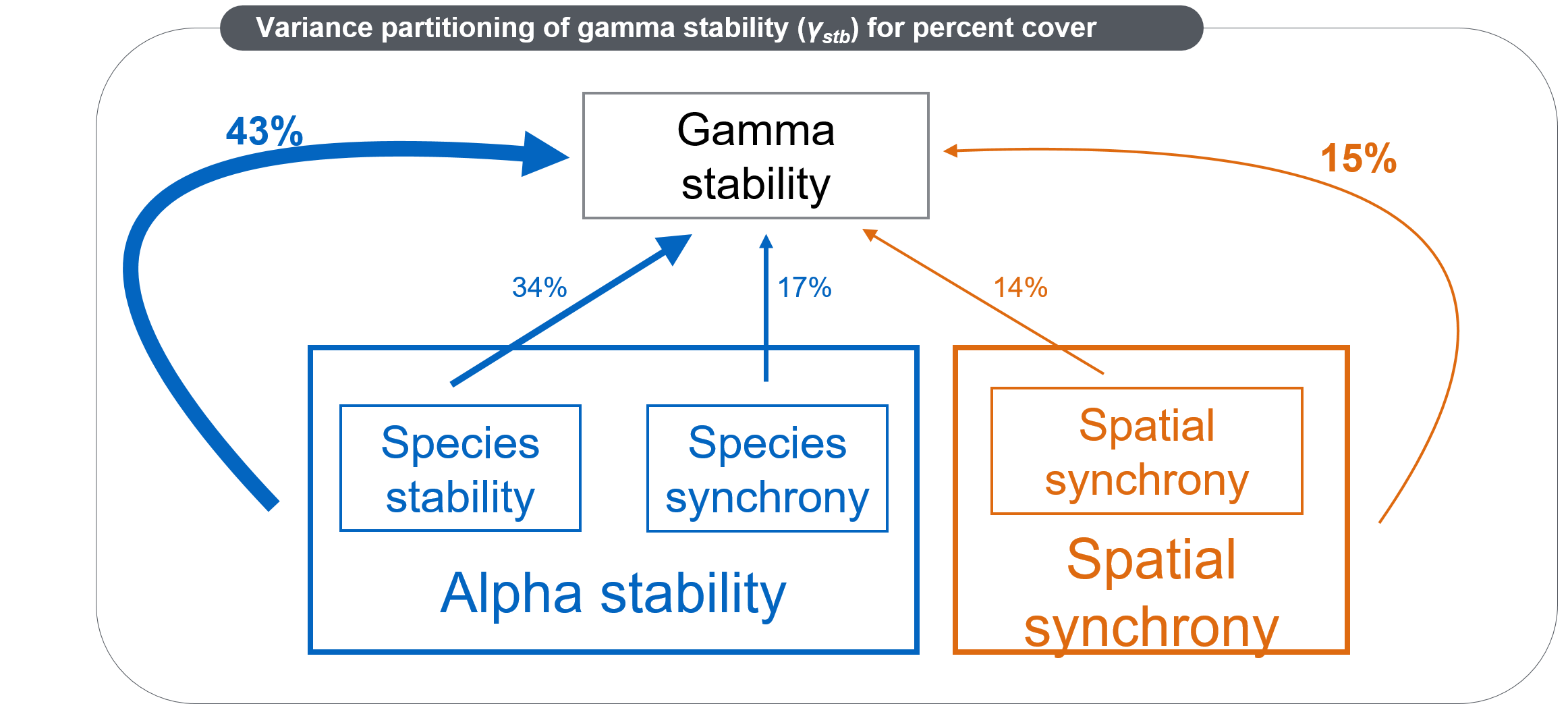


Figure S3. Variance partitioning results for gamma stability constrained to sites with 1m^2^ plots. Results are qualitatively similar to those reported in the main text (Fig. 2).


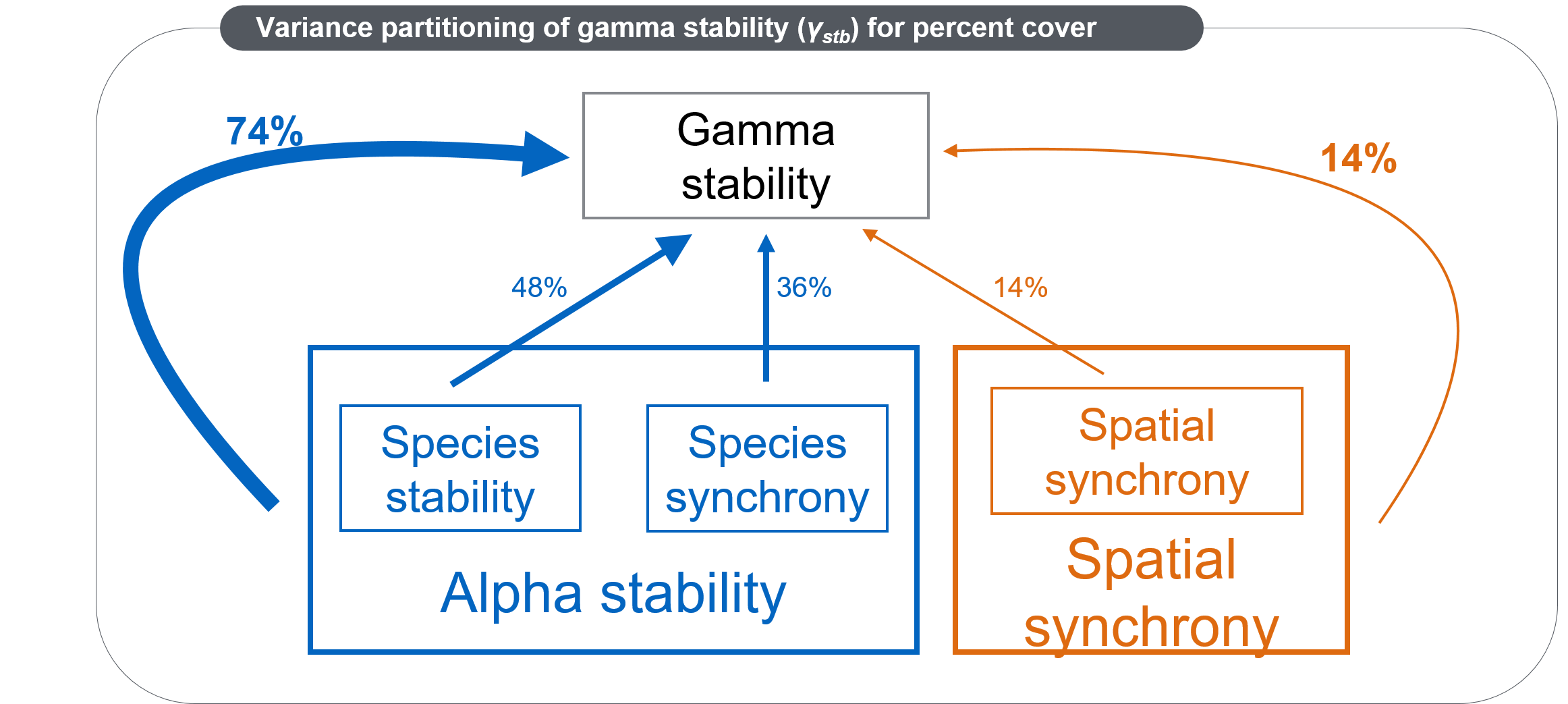


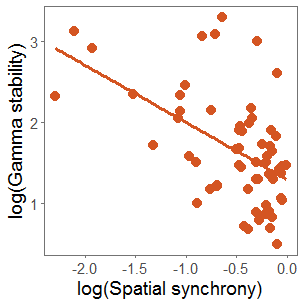


Figure S4. Variance partitioning results and bivariate relationship between spatial synchrony and gamma stability, removing one point having a large negative log(spatial synchrony value). The relationship is qualitatively similar to that with the point included. The bivariate relationship is significant at α=0.05 and the adjusted R^2^ is 0.29.


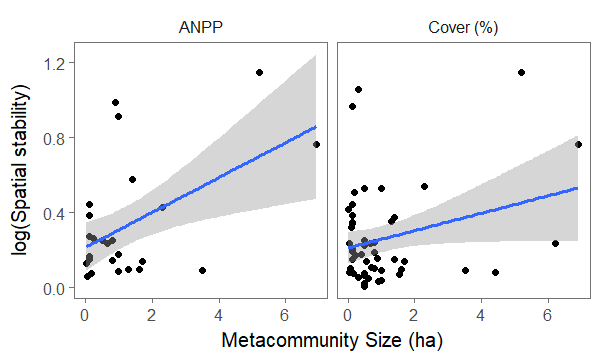


P=0.05

R^2^=0.07

P<0.01

R^2^=0.26

Figure S5. Regression between metacommunity size and spatial stabilization using aboveground net primary productivity (ANPP) and species abundance (Cover) data. Spatial stabilization factors were log transformed to satisfy normality assumptions.


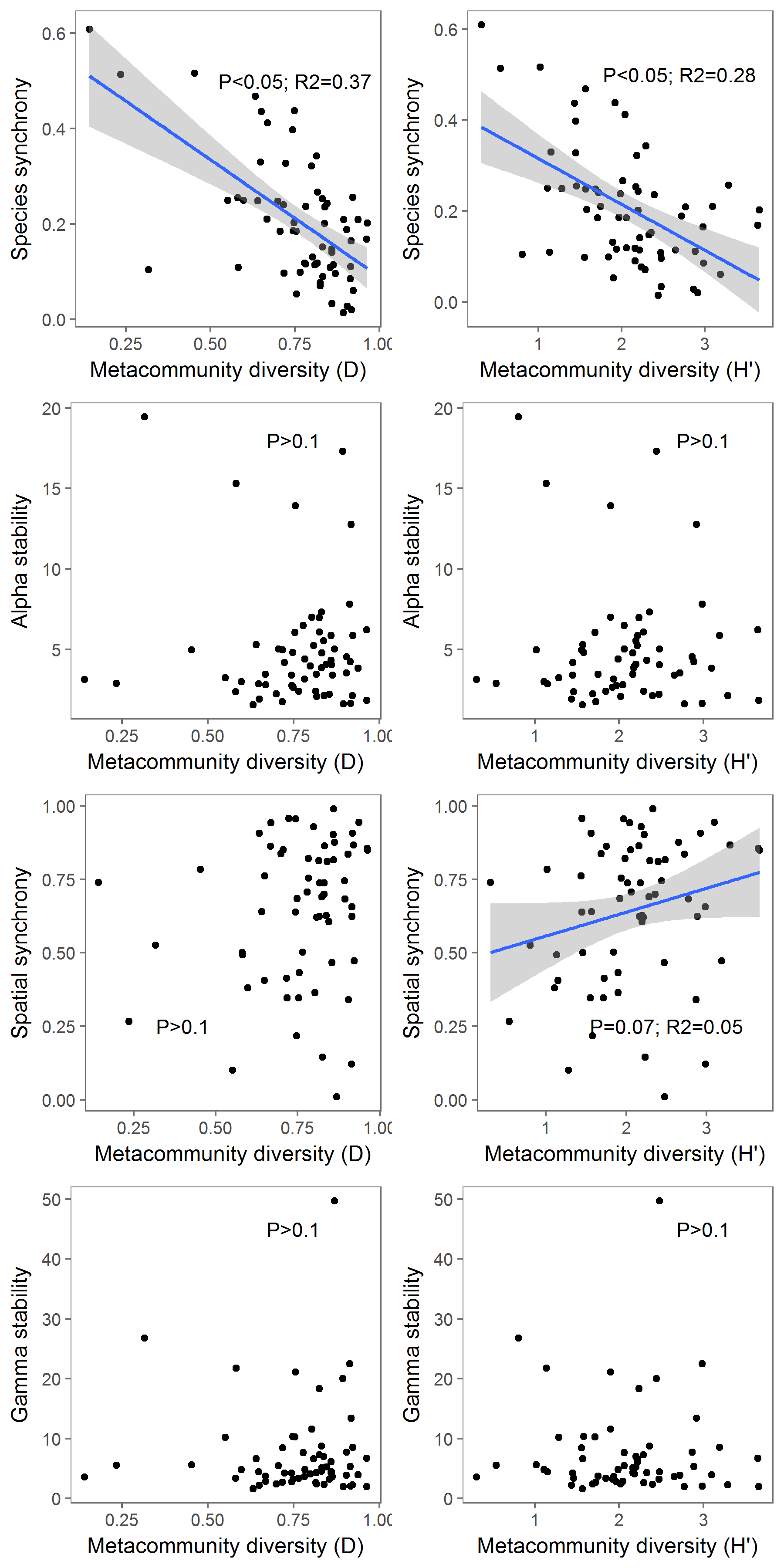


Figure S6. Bivariate regressions comparing metacommunity Simpson’s (left side) and Shannon’s (right) diversity versus stability and synchrony metrics of individual metacommunities. Trendlines are present only when regression is significant at α=0.1. Shaded area surrounding trendline represents standard error. Intercepts of significant regressions: Species synchrony-Simpson’s = 0.58 (s.e. = 0.06); Species synchrony-Shannon’s = 0.42 (0.05); Spatial synchrony-Shannon’s = 0.47 (0.10). Slopes of significant regressions: Species synchrony-Simpson’s = -0.49 (s.e. = 0.08); Species synchrony-Shannon’s = -0.10 (0.02); Spatial synchrony-Shannon’s = 0.08 (0.04).


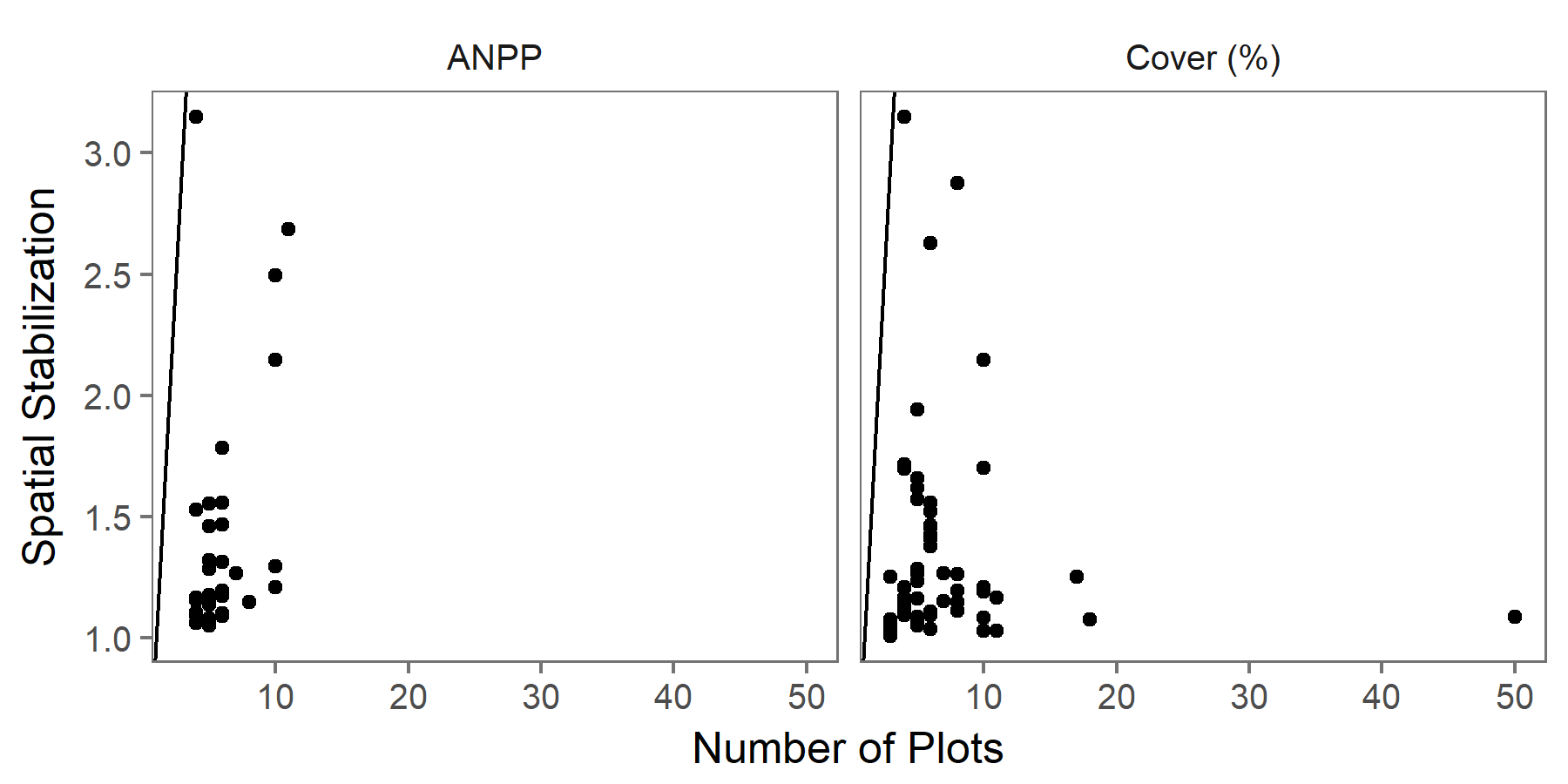


Figure S7.  Linkage between the number of plots in a metacommunity and the spatial stabilization factor of that metacommunity using aboveground net primary productivity (ANPP) and species cover data sets. The black line represents a limiting theoretical case where the spatial stabilization factor is driven completely by demographic stochasticity, and thus scales in direct proportion to the number of plots in the meta-community. We found no evidence of systematic bias introduced by the number of plots in the meta-community.


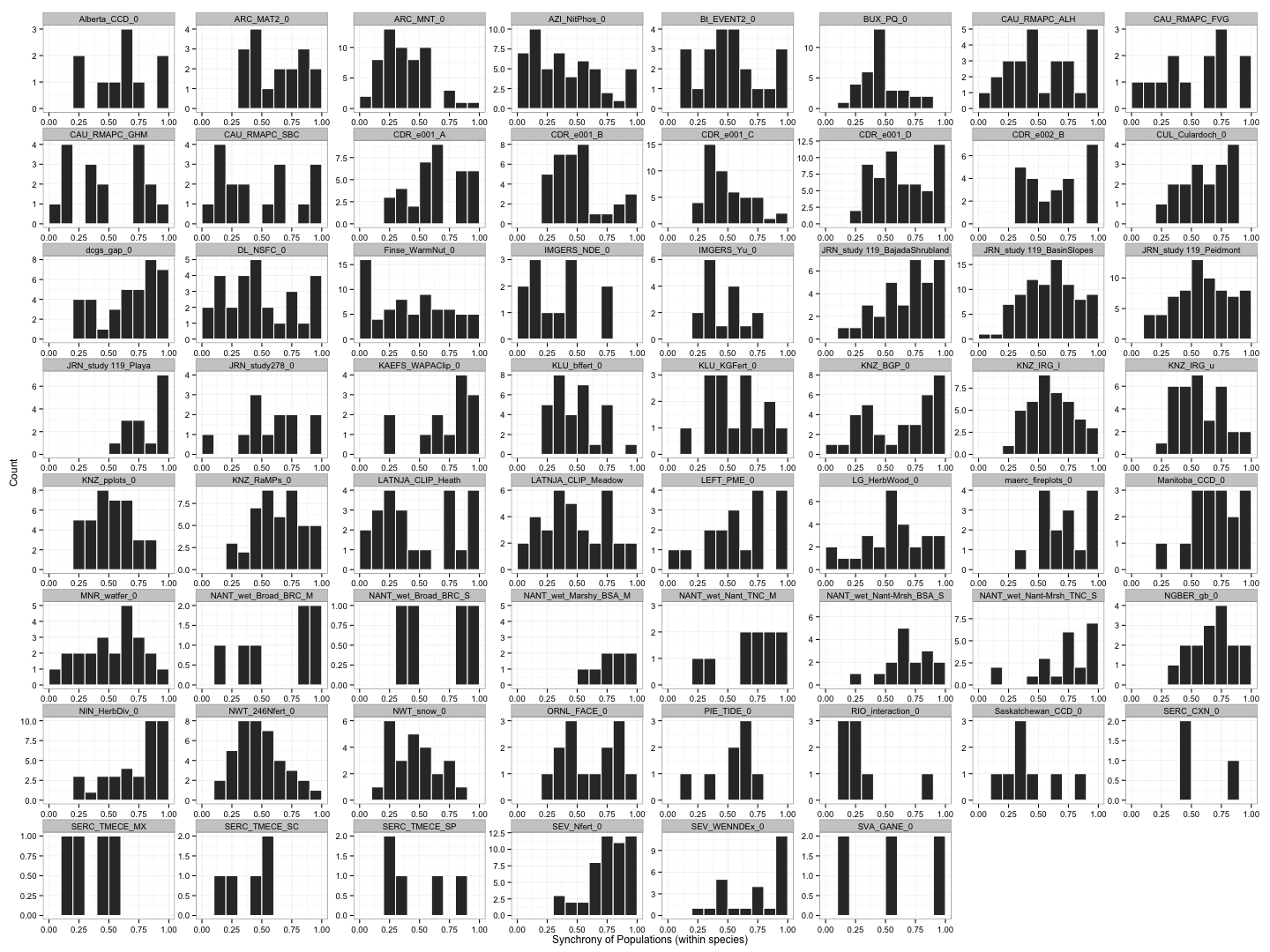

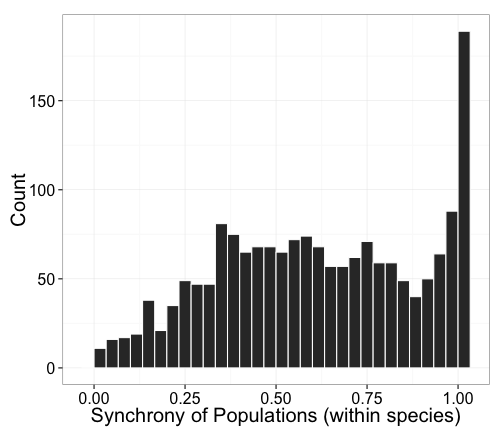


Figure S8. Histograms showing the frequency of within-species (population) synchrony levels among patches through time in individual community types (top) and from all community types (left). A synchrony of 1 means populations (i.e., species cover in plots) of a species are perfectly synchronous across plots through time. Values less than 1 indicate asynchrony among populations. All x-axis extend from zero to one.
