# Supplementary material for: Asynchrony among local communities stabilises ecosystem function of metacommunities
Source: Ecol Lett. 2017 Oct 24;20(12):1534–45. doi: 10.1111/ele.12861 (PMC6849522; doi:10.1111/ele.12861)
Supplement: Supplementary file 2 [file ELE-20-1534-s002.docx]

Table S 1. Site-level information for data sets used in analyses.

| **Continent** | **Country** | **Location** | **Data Source Paper** | **Ecosystem Type** | **MAP (mm)** | **ANPP (g/m^2^)** | **# of years** | **# of plots** | **Species level data measurement type** | **Meta-community size (ha)** |
| --- | --- | --- | --- | --- | --- | --- | --- | --- | --- | --- |
| Australia | Australia | Langi Ghiran State Park | Price and Morgan, Plant Ecology 2007, 29:37 | woodland | 588 | <15 | 3 | 10 | Presence/absence | <1 |
| Eurasia | China | Inner Mongolia Grassland Research Station | Yu et al. 2010, Ecology Letters, 13:1390-1399 | grassland | 345 | 135 | 8 | 6 | Biomass | 1.0 |
| Eurasia | China | Restoration Ecological Research Station, Duolon county | Xu et al. 2012, PloSOne, 7: e39762 | temperate steppe | 361 | 235 | 6 | 7 | ANPP | 0.66 |
| Eurasia | Germany | Ecological-Botanical Garden, University of Bayreuth | Walter et al. 2012 Ag. Ecosystems and Environment 148, 1-10 | semi-natural grassland | 724 | 559 | 5 | 5 | ANPP | 0.175 |
| Eurasia | Norway | Sandalsnuten, Finse | Klanderud 2008 Journal of Vegetation Science, 19: 363-372 | alpine | 1068 | 95 | 3 | 10 | Frequency | 0.12 |
| Eurasia | Norway | Svalbard | Madan et al. 2007 Polar Biology 30:559-570 | tundra | 385 | < 1.5 | 3 | 4 | Percent cover | 0.05 |
| Eurasia | Russia | Teberda Biosphere Reserve, NW Caucasus | Onipchenko et al. Plant Soil 2012. 357: 103-115 | alpine lichen heath | 1400 | 140 | 3 | 4 | Biomass | 3.5 |
| Eurasia | Russia | Teberda Biosphere Reserve, NW Caucasus | Onipchenko et al. Plant Soil 2012. 357: 103-115 | Festuca varia grassland | 1400 | 320 | 3 | 4 | Biomass | 5.2 |
| Eurasia | Russia | Teberda Biosphere Reserve, NW Caucasus | Onipchenko et al. Plant Soil 2012. 357: 103-115 | alpine Geranium-Hedysarum meadow | 1400 | 350 | 3 | 4 | Biomass | 1.6 |
| Eurasia | Russia | Teberda Biosphere Reserve, NW Caucasus | Onipchenko et al. Plant Soil 2012. 357: 103-115 | alpine snowbed community | 1400 | 150 | 3 | 4 | Biomass | 0.12 |
| Eurasia | Scotland | Culardoch Experimental Site, Eastern Highlands | Britton and Fisher, Journal of Applied Ecology, 2007, 44: 125-135 | alpine heathland | 1051 | 105 | 5 | 6 | Percent cover | 0.09 |
| Eurasia | Sweden | Latnjajaure Field Station | Alatalo et al. PeerJ, 2014 2: e406 | alpine heath | 808 | 383 | 3 | 8 | Percent cover | 0.4 |
| Eurasia | Sweden | Latnjajaure Field Station | Alatalo et al. Sci. Rep., 2015 5: 10197 | alpine meadow | 808 | 476 | 3 | 8 | Percent cover | 0.3 |
| Eurasia | UK | Health and Safety Laboratory, Harpur Hill, Derbyshire | Fridley et al. Global Change Biology, 2011, 17:2002-2011 | pasture | 1200 | 380 | 13 | 5 | Point intercept/Pin hits | 0.027* |
| North America | Canada | Gap Prairie Farm Rehabilitation Administration, Saskatchewan | White et al. Agronomy Journal, 2014, 106: 33-42 | northern temperate grassland | 262 | 132 | 3 | 5 | Percent cover | 0.2 |
| North America | Canada | Kluane Lake, Yukon | Turkington et al. Journal of Ecology 2002, 90: 325-337 | boreal forest understory | 230 | 145 | 10 | 8 | Percent cover | 6.2 |
| North America | Canada | Kluane Lake, Yukon | McLaren and Turkington 2010 Journal of Ecology, 98: 459-469 | northern temperate grassland | 230 | 175 | 6 | 5 | Biomass | 0.5 |
| North America | Canada | Riding Mountain National Park, Manitoba | White et al. Agronomy Journal, 2014, 106: 33-42 | northern temperate grassland | 349 | 282 | 3 | 5 | Percent cover | 0.2 |
| North America | Canada | Kinsella Research Ranch, University of Alberta | White et al. Agronomy Journal, 2014, 106: 33-42 | northern temperate grassland | 315 | 191 | 3 | 5 | Percent cover | 0.3 |
| North America | USA | Arctic LTER, Alaska | Gough et al. 2007, Oikos, 116: 407-418 | moist acidic tundra | 312 | 185 | 11 | 4 | Percent cover | 1.4 |
| North America | USA | Arctic LTER, Alaska | Gough and Hobbie, 2003, Oikos, 103:204-216 | moist non-acidic tundra | 312 | 150 | 9 | 3 | Percent cover | 0.6 |
| North America | USA | Cedar Creek LTER, Minnesota | Tilman Ecological Monographs, 1987, 57: 189-214 | tallgrass prairie | 826 | 277 | 24 | 6 | Biomass | 0.13 |
| North America | USA | Cedar Creek LTER, Minnesota | Tilman Ecological Monographs, 1987, 57: 189-214 | tallgrass prairie | 826 | 277 | 24 | 6 | Biomass | 0.13 |
| North America | USA | Cedar Creek LTER, Minnesota | Tilman Ecological Monographs, 1987, 57: 189-214 | tallgrass prairie | 826 | 277 | 30 | 6 | Biomass | 0.13 |
| North America | USA | Cedar Creek LTER, Minnesota | Tilman Ecological Monographs, 1987, 57: 189-214 | tallgrass prairie | 826 | 277 | 24 | 5 | Biomass | 0.13 |
| North America | USA | Cedar Creek LTER, Minnesota | Tilman Ecological Monographs, 1987, 57: 189-214 | tallgrass prairie | 826 | 277 | 19 | 6 | Biomass | 0.13 |
| North America | USA | Chequamegon-Nicolet National Forest, Wisconsin | Kern et al. Journal of Plant Ecology, 2012 6:101-112 | temperate mixed wood forest understory | 779 | 708 | 4 | 6 | Percent cover | 0.8 |
| North America | USA | Jornada LTER, Arizona | Mun and Whitford 1989 Plant and Soil 120: 225-231 | desert, Bajada Shrubland | 269 | 104 | 5 | 11** | Percent cover | 0.9 |
| North America | USA | Jornada LTER, Arizona | Mun and Whitford 1989 Plant and Soil 120: 225-231 | desert, Basin Slopes | 269 | 113 | 5 | 50** | Percent cover | 4.41 |
| North America | USA | Jornada LTER, Arizona | Mun and Whitford 1989 Plant and Soil 120: 225-231 | desert, Piedmont | 269 | 79 | 5 | 18** | Percent cover | 1.53 |
| North America | USA | Jornada LTER, Arizona | Mun and Whitford 1989 Plant and Soil 120: 225-231 | desert, Playa | 269 | 199 | 5 | 7** | Percent cover | 0.54 |
| North America | USA | Jornada LTER, Arizona | Reichmann et al. 2013 Ecology 34: 435-443 | desert | 264 | 173 | 3 | 18 | Line intercept | 144 |
| North America | USA | Kessler Atmospheric and Ecological Field Station, Oklahoma | Xu et al. 2013, Global Change Biology, 19: 2753-2764 | tallgrass prairie | 782 | 363 | 4 | 4 | Point intercept/Pin hits | 0.07 |
| North America | USA | Konza LTER, Kansas | Eom et al. 1999 The American Midland Naturalist, 142: 55-70 | tallgrass prairie | 833 | 433 | 5 | 4 | Percent cover | 2.3 |
| North America | USA | Konza LTER, Kansas | Knapp et al. 2001 Ecosystems 4:19-28 | tallgrass prairie, lowland | 835 | 441 | 19 | 5 | Percent cover | 0.8 |
| North America | USA | Konza LTER, Kansas | Knapp et al. 2001 Ecosystems 4:19-29 | tallgrass prairie, upland | 835 | 506 | 19 | 4 | Percent cover | 0.8 |
| North America | USA | Konza LTER, Kansas | Avolio et al. 2014, Journal of Ecology, 102: 1649-1660 | tallgrass prairie | 835 | 484 | 11 | 6 | Percent cover | 0.12 |
| North America | USA | Konza LTER, Kansas | Knapp et al. Science 2002, 298:2202-2205 | tallgrass prairie | 835 | 600 | 15 | 6 | Percent cover | 1 |
| North America | USA | Macarthur Agro-Ecological Research Center, Florida | Boughton et al. 2013, Biological Conservation 158: 239-247 | pasture | 1365 | 967 | 8 | 4 | Percent cover | 0.48 |
| North America | USA | McLaughlin Natural Reserve, California | Eskelinen & Harrison 2015, PNAS 112: 13009-13014 | annual grassland | 697 | 613.92 | 3 | 11 | Percent cover | 0.89 |
| North America | USA | Nanticoke River watershed, MD & DE | Baldwin, 2013, Esuaries and Coasts. 36: 547-558 | tidal freshwater wetland | 1199 | 463 | 4 | 3 | Percent cover | 0.5 |
| North America | USA | Nanticoke River watershed, MD & DE | Baldwin, 2013, Esuaries and Coasts. 36: 547-558 | tidal freshwater wetland | 1199 | 463 | 4 | 3 | Percent cover | 0.5 |
| North America | USA | Nanticoke River watershed, MD & DE | Baldwin, 2013, Esuaries and Coasts. 36: 547-558 | tidal freshwater wetland | 1199 | 463 | 4 | 3 | Percent cover | 0.5 |
| North America | USA | Nanticoke River watershed, MD & DE | Baldwin, 2013, Esuaries and Coasts. 36: 547-558 | tidal freshwater wetland | 1199 | 463 | 4 | 3 | Percent cover | 0.5 |
| North America | USA | Nanticoke River watershed, MD & DE | Baldwin, 2013, Esuaries and Coasts. 36: 547-558 | tidal freshwater wetland | 1199 | 463 | 4 | 3 | Percent cover | 0.5 |
| North America | USA | Nanticoke River watershed, MD & DE | Baldwin, 2013, Esuaries and Coasts. 36: 547-558 | tidal freshwater wetland | 1199 | 463 | 3 | 3 | Percent cover | 0.5 |
| North America | USA | Niwot LTER, Colorado | Bowman et al. 2006 Ecological Applications 16: 1183-1193 | alpine | 993 | 148 | 8 | 5 | Percent cover | 0.041 |
| North America | USA | Niwot LTER, Colorado | Smith et al. 2012 Arctic Antarctic and Alpine Research, 44: 135-142 | alpine | 993 | 240 | 7 | 6 | Percent cover | 0.024* |
| North America | USA | Northern Great Basin Experimental Range, Oregon | Bates et al. Journal of Arid Environments 2006 64: 670-697 | sagebrush steppe | 300 | 80 | 6 | 4 | Percent cover | 0.14* |
| North America | USA | Oak Ridge National Environmental Reserch Park, Tennessee | Souza et al. 2010. Journal of Plant Ecology, 3: 33-39 | forest understory | 1390 | 170 | 4 | 8 | Biomass | 1.7 |
| North America | USA | Plum Island Estuary, Massachusets | Deegan et al. 2012 Nature, 490:388-392 | salt marsh | 1277 | 1118 | 8 | 6 | Percent cover | 1.3 |
| North America | USA | Sevilleta LTER, New Mexico | Ladwig et al. 2012 Oecologia, 169: 177-185 | desert grassland | 250 | 120 | 9 | 10 | ANPP | 0.14 |
| North America | USA | Sevilleta LTER, New Mexico | Collins et al. 2017 Global Change Biology | desert grassland | 250 | 120 | 6 | 5 | ANPP | 0.4 |
| North America | USA | Wichita State Ninnescah Reserve, Oklahoma | Unpublished | tallgrass prairie | 788 | 721 | 4 | 8 | Percent cover | 0.71 |
| South America | Argentina | Rio Mayo Experimental Station | Yahdijian and Sala, Ecology, 2006, 87: 952-962 | grassland | 168 | 52.27 | 4 | 10 | Percent cover | 1.0 |
| Eurasia | China | the Research Station of the Alpine Meadow and Wetland Ecosystems of Lanzhou University | Zhang et al. 2015, Scientific Reports, 5: 16832 | alpine meadow | 620 | 440 | 3 | 6 | Percent cover | 1.4 |
| Eurasia | China | Inner Mongolia Grassland Ecosystem Research Station | Zhang et al. GCB, 2016, 22: 1446-1455 | temperate grassland | 351.4 | 216.4 | 3 | 10 | ANPP | 6.9 |
| North America | USA | Lefthand Canyon, Colorado | Prevéy and Seastedt, Journal of Ecology, 2014, 102: 1549-1561 | semi-arid grassland | 475 | 198.4 | 3 | 10 | Point intercept/Pin hits | 0.23 |
| North America | USA | Smithsonian Environmental Research Center | Langley et al. in: Lang, Macintyre, and Rützler (eds.) Proc. Smithsonian Marine Sciences Symposium, No. 38 pg 391-400 | tidal saltmarsh | 1064 | 621.4 | 10 | 5 | ANPP | 0.2 |
| North America | USA | Smithsonian Environmental Research Center | Curtis et al. Oecologia, 1989, 78: 20-26 | tidal saltmarsh - mixed | 1064 | 796.6 | 17 | 5 | Stem density | 0.1 |
| North America | USA | Smithsonian Environmental Research Center | Curtis et al. Oecologia, 1989, 78: 20-26 | tidal saltmarsh - Scirpus | 1064 | 877.4 | 17 | 5 | Stem density | 0.04 |
| North America | USA | Smithsonian Environmental Research Center | Curtis et al. Oecologia, 1989, 78: 20-26 | tidal saltmarsh - Spartina | 1064 | 787.9 | 17 | 5 | Stem density | 0.07 |
| * Metacommunity area was estimated by summing the area of individual blocks/plots within a study site. This was only done when we were unable to obtain the size of the area all plots were located in from the literature | | | | | | | | | | |
| ** Not all plots are statistically independent | | | | |  |  |  |  |  |  |

|  | ANPP and cover | | ANPP | | Cover | |
| --- | --- | --- | --- | --- | --- | --- |
| Statistic | Full database | 1m^2^ plots | Full database | 1m^2^ plots | Full database | 1m^2^ plots |
| Mean | 1.4 | 1.4 | 1.5 | 1.5 | 1.3 | 1.4 |
| Median | 1.2 | 1.2 | 1.3 | 1.2 | 1.2 | 1.2 |
| Min | 1.01 | 1.01 | 1.1 | 1.09 | 1.01 | 1.01 |
| Max | 3.2 | 2.9 | 3.2 | 2.7 | 3.2 | 2.9 |

Table S2. Summary statistics of spatial stabilization factors across metacommunities. Summary statistics are for all metacommunities (Full database) and including only metacommunities having 1m2 plots. Summary statistics are shown combining aboveground net primary productivity (ANPP) and species abundance data, ANPP data alone, and species abundance data alone.

Table S3. Output from linear regressions comparing cross-metacommunity patterns of spatial and population synchrony with the number of plots within a metacommunity (plotNum), the number of years of data present (yearsNum), and the size of plots within the metacommunity (plotSize). Data points with high influence were discarded from the analysis as not to bias findings by a small subset of sites. P values <0.1 are italicized.

|  | Spatial synchrony | | | | | Population synchrony | | | | |
| --- | --- | --- | --- | --- | --- | --- | --- | --- | --- | --- |
| Effect | test stat | pval | df | n | r.squared | test stat | pval | df | n | r.squared |
| plotNum | 0.0016474 | 0.967764 | 2 | 60 | <0.01 | 0.593534 | 0.444183 | 2 | 60 | 0.01 |
| yearsNum | 1.2900277 | 0.2607144 | 2 | 60 | 0.02 | 0.018303 | 0.892852 | 2 | 60 | <0.01 |
| plotSize | 0.8113861 | 0.3715684 | 2 | 60 | 0.01 | 0.574014 | 0.451845 | 2 | 60 | 0.01 |

Table S4. Statistical model output from regressions comparing average plot level Shannon’s species diversity for a metacommunity with the species synchrony, alpha stability, and gamma stability of a metacommunity. Species synchrony and alpha stability values were averaged across plots to obtain one number for each metacommunity.

| Comparison | Df (num,den) | F value | P value | R2 |
| --- | --- | --- | --- | --- |
| Sp. synchrony ~ Shannon’s | 2,60 | 15.19 | <0.01 | 0.20 |
| α Stability ~ Shannon’s | 2,60 | 3.46 | 0.07 | 0.02 |
| γ Stability ~ Shannon’s | 2,60 | 1.21 | 0.28 | 0.02 |

|  | Shannon’s (H′) | | | | Simpson’s (D) | | | |
| --- | --- | --- | --- | --- | --- | --- | --- | --- |
| Effect | test stat | pval | df | r.squared | test stat | pval | df | r.squared |
| Species synchrony | 23.3 | <0.01 | 60 | 0.28 | 34.94 | <0.01 | 60 | 0.37 |
| Alpha stability | 0.29 | 0.59 | 60 | <0.01 | 0.40 | 0.53 | 60 | <0.01 |
| Spatial synchrony | 3.41 | 0.07 | 60 | 0.05 | 1.91 | 0.17 | 60 | 0.03 |
| Gamma stability | 0.03 | 0.87 | 60 | <0.01 | 0.02 | 0.89 | 60 | <0.01 |

Table S5. Statistical model output from regressions comparing metacommunity Shannon’s and Simpson’s species diversity with species synchrony, alpha stability, spatial synchrony, and gamma stability of a metacommunity. Species synchrony and alpha stability values were averaged across plots to obtain one number for each metacommunity. Metacommunity diversity was calculated from species composition of the entire metacommunity, obtained by averaging species covers across all plots within a metacommunity.
